# Salvianolic Acid A, a Novel Matrix Metalloproteinase-9 Inhibitor, Prevents Cardiac Remodeling in Spontaneously Hypertensive Rats

**DOI:** 10.1371/journal.pone.0059621

**Published:** 2013-03-22

**Authors:** Baohong Jiang, Defang Li, Yanping Deng, Fukang Teng, Jing Chen, Song Xue, Xiangqian Kong, Cheng Luo, Xu Shen, Hualiang Jiang, Feng Xu, Wengang Yang, Jun Yin, Yanhui Wang, Hui Chen, Wanying Wu, Xuan Liu, De-an Guo

**Affiliations:** 1 Shanghai Institute of Materia Medica, Chinese Academy of Sciences, Shanghai, China; 2 College of Traditional Chinese Medicine, China Pharmaceutical University, Nanjing, China; 3 Shenyang Pharmaceutical University, Shenyang, China; 4 Department of Cardiovascular Surgery, Renji Hospital, Shanghai Jiaotong University School of Medicine, Shanghai, China; Medical College of Wisconsin, United States of America

## Abstract

Cardiac fibrosis is a deleterious consequence of hypertension which may further advance to heart failure and increased matrix metalloproteinase-9 (MMP-9) contributes to the underlying mechanism. Therefore, new therapeutic strategies to attenuate the effects of MMP-9 are urgently needed. In the present study, we characterize salvianolic acid A (SalA) as a novel MMP-9 inhibitor at molecular, cellular and animal level. We expressed a truncated form of MMP-9 which contains only the catalytic domain (MMP-9 CD), and used this active protein for enzymatic kinetic analysis and Biacore detection. Data generated from these assays indicated that SalA functioned as the strongest competitive inhibitor of MMP-9 among 7 phenolic acids from *Salvia miltiorrhiza*. In neonatal cardiac fibroblast, SalA inhibited fibroblast migration, blocked myofibroblast transformation, inhibited secretion of intercellular adhesion molecule (ICAM), interleukin-6 (IL-6) and soluble vascular cell adhesion molecule-1 (sVCAM-1) as well as collagen induced by MMP-9 CD. Functional effects of SalA inhibition on MMP-9 was further confirmed in cultured cardiac H9c2 cell overexpressing MMP-9 *in vitro* and in heart of spontaneously hypertensive rats (SHR) *in vivo*. Moreover, SalA treatment in SHR resulted in decreased heart fibrosis and attenuated heart hypertrophy. These results indicated that SalA is a novel inhibitor of MMP-9, thus playing an inhibitory role in hypertensive fibrosis. Further studies to develop SalA and its analogues for their potential clinical application of cardioprotection are warranted.

## Introduction

Myocardial fibrosis is a common pathological feature seen in many patients with hypertension and is hypothesized to be the final common pathway that ultimately results in irreversible heart failure [1]. Cardiac fibrillar collagen network in the extracellular matrix (ECM) provides the structural support for cardiomyocytes and coronary vessels, contributing to maintain the cardiac diastole and systole function. Hypertension, as one pathological stimulus to this network, leads to the development of cardiac remodeling characterized by rearrangement of cardiac components including ECM proteins [2].

Matrix metalloproteinases (MMP) represent an important biological system within the myocardium designed to maintain the complex and dynamic microenvironment of the ECM [3]. Recent studies have examined the important role of MMPs in the cardiac remodeling associated with hypertension. Results from clinical studies involving hypertensive patients demonstrated that the serum levels and activities of MMP-2, MMP-9 were increased in hypertensive patients with diastolic heart failure compared to those without diastolic heart failure, which may reflect abnormal ECM metabolism in hypertension [4], [5]. MMP-9 knockout mice showed increased myocardial protection and attenuated remodeling after experimental acute myocardial infarction [6]. The upstream and downstream pathway of MMP-9 on cardiac protection was elucidated. MMP-9 deletion attenuated the age-related decline in diastolic function, in part by reducing TGF-β signaling-induced periostin and CTGF expression. The stress-inducible transcriptional regulator p8 was increased in failing human hearts and was required for MMP-9 induction on cardiac fibroblasts [7]. And also chymase-dependent MMP-9 activation was important in the pathophysiology of myocardial infarction reperfusion and fibrosis [8].

*Salvia miltiorrhiza*, one of the most important traditional herbal medicines, has been widely used in clinic in China for the treatment of cardiovascular diseases [9]. The major water-soluble chemical constituents of *Salvia miltiorrhiza* include salvianolic acid A (SalA), salvianolic acid B (SalB), salvianolic acid C (SalC), lithospermic acid, rosmarinic acid, danshensu, and protocatechualdehyde [10]. Our previous study demonstrated that SalB inhibited matrix MMP-9 activity in rat heart with myocardial infarction and ameliorated cardiac remodeling [11]. To further develop more effective MMP-9 inhibitor than SalB, we screen phenolic acids in *Salvia miltiorrhiza* and found SalA is a more potential candidate.

Firstly, we set to establish biochemical evidences that SalA directly binds to MMP-9 and competitively inhibits its gelatinase activity using purified recombinant MMP-9 CD by surface plasmon resonance analysis and enzyme kinetic analysis. To elucidate the effects of SalA on the function of fibroblast treated with MMP-9 CD, migration, proliferation, myofibroblastic phenotype and secretion of cytokines were examined by transwell assay, CCK-8 assay, immunofluorescence of α-SMA and procarta cytokine profiling assay, respectively. A stable H9c2 cell lines overexpressing MMP-9 was set up and the inhibitory effects of SalA on MMP-9 was further confirmed. Finally, the *in vivo* activity of SalA on MMP-9 inhibition and anti-cardiac fibrosis was detected using SHR rat. A model for the mechanism of SalA against cardiac remodeling was constructed. To sum up, we identified SalA as a novel MMP-9 inhibitor, evaluated the antifibrotic activity and elucidated the underlying mechanism of SalA.

## Materials and Methods

### Cell Line, Animal and Materials

Rat heart cell line H9c2 cells (Catalog No. GNR 5) were purchased from Cell Bank of China Science Academy (Shanghai, China). All media and culture reagents were products of Gibco (Grand Island, NY) unless specified otherwise. Two months old male SHR rats were purchased from Shanghai Center of Experimental Animals and acclimatized in temperature and humidity-controlled rooms with a 12-h dark/light cycle throughout the study. All procedures involving animals were approved by the Institutional Animal Care and Use Committee at Shanghai Institute of Materia Medica (IACUC number: SIMM-AE-GDA-2010-06). National Institutes of Health (NIH) Guide for the Care and Use of Laboratory Animals was followed throughout. SalA, SalB, SalC, rosmarinic acid, lithospermic acid, pyrocatechol and sodium danshensu were purchased from Shanghai Yousi Bio-Tech Co., Ltd. Purity of SalA and other phenolic acids were analyzed by high performance liquid chromatography and confirmed to be more than 99% (Fig. S 1). For detailed methods and reagent sources, see Supplementary material.

### Real Time Binding Measured by Surface Plasmon Resonance

DNA fragments encoding the catalytic domain of MMP-9 (MMP-9 CD, corresponding to residues 107–216 and 391–444) were constructed and subcloned into a pET-15b vector (Novagen). The purified recombinant MMP-9 CD displayed significant gelatinase activity detected by zymography assay (Fig. S2), and the active MMP-9 CD was then used to detect the binding activity of SalA to MMP-9 as our previous report [11]. Surface plasmon resonance analysis was performed using Biacore 3000 (Biacore AB). In brief, MMP-9 CD protein (5.8 µmol/L) in 10 mmol/L sodium acetate buffer (pH 3.73) was covalently immobilized on the hydrophilic carboxymethylated dextran matrix of the CM5 sensor chip (BIAcore AB) using standard primary amine coupling procedure. Phenolic acid at various concentrations in HBS-EP running buffer (10 mmol/L HEPES pH 7.4, 150 mmol/L NaCl, 3.4 mmol/L EDTA and 0.005% surfactant P20) were injected at a constant flow rate of 30 µL/min at 25°C. All data were analyzed by BIAevaluation software and the sensorgrams were processed by automatic correction for nonspecific bulk refractive index effects.

### MMP-9 Inhibition Assay

Phenolic acids at the indicated concentrations were incubated with the reaction mixtures contained MMP-9 CD (15 nmol/L) and 5,5′-dithiobis-(2-nitrobenzoic acid) (1 mmol/L) at 4°C for 30 min. The reaction was initiated by the addition of thiopeptolide (Ac-Pro-Leu-Gly-S-Leu-Leu-Gly-OEt). Hydrolysis of thiopeptolide was monitored at 412 nm using Benchmark Plus™ microplate spectrophotometer (Bio-Rad) at room temperature. Kinetic analysis of phenolic acids against MMP-9 CD was calculated using double reciprocal plots of 1/V versus 1/[thiopeptolide]. The slope of every double reciprocal plot is the K_m_^app^/V_max_ of enzyme at different compound concentration. Secondary plot was drawn through K_m_^app^/V_max_ versus compound concentration. K_m_^app^ is apparent value of K_m_; K_i_ is inhibition constant; K_i_ was calculated using the equation K_i_ = [K_m_/V_max_][compound]/(K_m_^app^/V_max_ –K_m_/V_max_).

### Neonatal Cardiac Fibroblast Culture

Primary cardiac fibroblast was obtained from neonatal rats and National Institutes of Health (NIH) Guide for the Care and Use of Laboratory Animals was followed throughout. Wistar rats (2–4 day old) were sacrificed by cervical dislocation; ventricles were minced and digested with 0.1% trypsin at 37°C for 8 min. The liberated cells were collected by centrifugations; non-digested chunk materials were further digested in 0.1% trypsin for an additional 8 min. This digestion procedure was repeated 3–4 times. All the liberated cells were pelleted by centrifugation at 1,000 rpm for 10 min, re-suspended in DMEM containing 10% fetal bovine serum (FBS), and cultured in a 100-mm noncoated culture flask at 37°C with 5% CO_2_ for 2 h. Adherent cardiac fibroblasts were collected and maintained in DMEM supplemented with 10% FBS, 100 U/mL penicillin, 100 µg/mL streptomycin [12]. Cardiac fibroblasts were treated by vehicle (Con); 200 nmol/L MMP-9 CD (MMP-9); 200 nmol/L MMP-9 CD plus 1 µmol/L SalA [MMP-9/SalA(1)]; or 200 nmol/L MMP-9 CD plus 10 µmol/L SalA [MMP-9/SalA(10)] for the following research. All the detections were conducted after 24 h treatment.

### Cell Migration and Proliferation Assays

Transwell assay was employed to evaluate effects of SalA on neonatal cardiac fibroblast migration induced by MMP-9 CD. Twenty thousand cells in 200 µL culture medium containing various concentrations of SalA were loaded onto PET track-etched membrane in modified Boyden chambers (BD Falcon Cell Culture Insert, BD), and same medium containing MMP-9 CD (200 nmol/L) was added to the lower side of the migration chamber. After incubating for 24 h, migrated cells (in the lower side of migration chamber) were stained with 0.1% crystal violet and counted with a microscope at 200 X magnification. Five to six view fields were counted on each membrane. Migration is expressed as the mean value of total number of migrated cells per field.

Cell proliferation assay was performed using Cell Counting Kit (CCK-8 kit, Dojindo Laboratory, Japan) as described previously [13]. Absorbance was detected at 450 nm (reference 650 nm) using a microplate reader (Tecan GENios, Austria).

### Detection of Myofibroblast Transformation

α-SMA expression was used to evaluate the effect of SalA on myofibroblast transformation from fibroblast induced by MMP-9 CD. Neonatal cardiac fibroblasts were cultured on glass slides until reaching ∼70% confluence. Cells were then stimulated with MMP-9 CD for 24 h, in the presence or absence of SalA, and fixed with 95% ethanol after extensive washing with PBS. Following an incubation with 0.3% H_2_O_2_ for 15 min, cells were permeabilized using 0.5% Triton-100. The slides were then incubated with a blocking solution containing 2% serum at 37°C for 30 min, and stained with a rabbit polyclonal anti α-SMA antibody (1∶50, Boster, China) for 1 h at room temperature, followed by incubation with a fluorescein-isothiocyanate-conjugated (FITC) anti-mouse second antibody (1∶50; DingGuo, China) and 5 µg/mL DAPI for 40 min at room temperature. Images were acquired by Olympus BX51 microscope plus Olympus DP71 CCD camera.

### Multiplex Cytokine Assay and Quantification of Collagen Synthesis

Procarta cytokine profiling kit (Panomics, CA, USA) was used to simultaneously detect 3 different rat cytokines in the cell culture supernatants, according to manufacturer’s instruction. Briefly, 50 µL antibody beads were added to each well of the Filter plate, washed with Wash buffer. Fifty microliter cell culture supernatant was then added to each well, incubated for at least 1 h at room temperature, and washed with Wash buffer. Afterwards, 25 µL per well of the Detection Antibody was added and the Filter plate was shaken at 500 rpm for 30 min at room temperature. After adding Sreptavidin-PE, the signals were detected using a Luminex 200 instrument (Bio-Rad, CA, USA). The cytokines included in the present study were intercellular adhesion molecule (ICAM), interleukin-6 (IL-6), tumor necrosis factor-α (TNF-α and soluble vascular cell adhesion molecule-1 (sVCAM-1).

Soluble collagen secreted by neonatal cardiac fibroblast was measured using picrosirius red assay with minor modification [14], [15]. Briefly, cells were cultured at 5×10^4^/mL cells in 6 well plates, and treated by vehicle, MMP-9 CD, MMP-9 CD plus SalA for 24 h. Culture medium (750 µL) were added to 1 mL 0.1% picrosirius red in saturated picric acid and stained for 1 h. The supernatant was removed by centrifugation at 12000 rpm for 10 minutes, and the pelleted dye was solubilized in 300 µL NaOH (0.1 M). 200 µL solution was transferred to 96 well plate for measuring absorbance at 595 nm using a UV spectrophotometer (Tecan GENios, Austria).

### H9c2 Cell Culture and MMP-9 Overexpression

DNA cloning using Gateway technology was carried out according to manufacturer’s instructions. Full-length MMP-9 cDNA (MGC-12688) was first subcloned into entry vector pDONR^TM^221, followed by recombination reaction between the *att*L-containing pDONR^TM^221 vector and *att*R-containing destination pDEST vector (Invitrogen, Carlsbad, CA, USA). The resulting expression vector pDEST-MMP-9 was transfected into H9c2 (Cell bank of Chinese Academy of Sciences, Shanghai; Catalog No. GNR 5) using Lipofectamine™ (Invitrogen, Carlsbad, CA). Stable H9c2 cell line was generated by selection with 2 mg/mL G418 (denoted as pDEST-MMP9) and maintained in 0.75 mg/mL G418, and expression of MMP-9 was evaluated using zymography assay. The pDEST vector without the MMP-9 insert also was transfected into H9c2 and the resulting stable cells were used as a control (denoted as pDEST). Cell morphology was captured using Olympus BX51 microscope in combination with an Olympus DP71 CCD camera.

### In-gel Gelatin Zymography

The enzymatic activities of H9c2 cells or tissue samples were detected by in-gel gelatin zymography. In brief, twenty µg protein from tissue or twenty microliter culture supernatant of H9c2 cells with different treatments was electrophoresed in 10% SDS-PAGE containing 1% gelatin as MMP substrate under non-reducing conditions. After electrophoresis, the gel was washed in 1% Triton X-100 for 1 h, rinsed in water, and incubated overnight in substrate buffer containing 50 mmol/L Tris-HCl, 5 mmol/L CaCl_2_, and 150 mmol/L NaCl (pH 7.5) at 37°C with gentle shaking. Then, the gel was stained in 0.1% Coomassie Blue R-250 and destained using 10% methanol, 5% acetic acid solution. The gel was scanned using a MiniBis system (DNR Bio-Imaging Systems Ltd) and the detected protein species corresponding to the expected molecular weight of MMP-9 was visualized.

### SalA Treatment on SHR

2-month old male SHR rats were given rodent chow containing 8% NaCl 4 weeks before SaA treatment to the end of the experiment. Rats were divided into four groups (20 per group) and were injected intraperitoneally with saline or SalA everyday at the indicated dose for 4 weeks. The four groups were named as SalA (0 mg/kg), SalA (2.5 mg/kg), SalA (5 mg/kg), SalA (10 mg/kg) basing on the dose of SalA, respectively.

### Histopathological Detection for SHR

After ketamine-xylazine anesthesia (ketamine: xylazine, 60∶5 mg/kg, intramuscularly), heart, liver and kidney samples from experimental animals were fixed with 4% neutral-buffered paraformaldehyde for 24 h. Specimens were paraffin-embedded, sliced at 5 µm, and stained with haematoxylin and eosin. After staining, the sections were rinsed with distilled water, dehydrated, and mounted with Permount. Photomicrographs were taken using an Olympus BX51 microscope plus Olympus DP71 CCD camera (Olympus Corporation). Software Image-Pro Plus version 6.0 was used and approximately 150 cells per heart were measured to determine the average myocyte circumference. Paraffin-embedded slices were also stained with 0.1% picric sirius red (Sigma-Aldrich Inc, St Louis, USA) for fibrillar collagen. Collagen volume fraction (CVF) was expressed as a percentage of the total area of the field occupied by collagen staining.

### Statistic Analysis

All quantitative values are given as mean ± S.E. Mean values of data from different treatment groups were compared using one-way ANOVA. After confirming the equal variances, least-significant difference (LSD) was used to compare the difference between groups. *P*<0.05 was considered to be statistically significant.

## Results

### Direct Interaction of SalA on MMP-9

**Figure 1:**
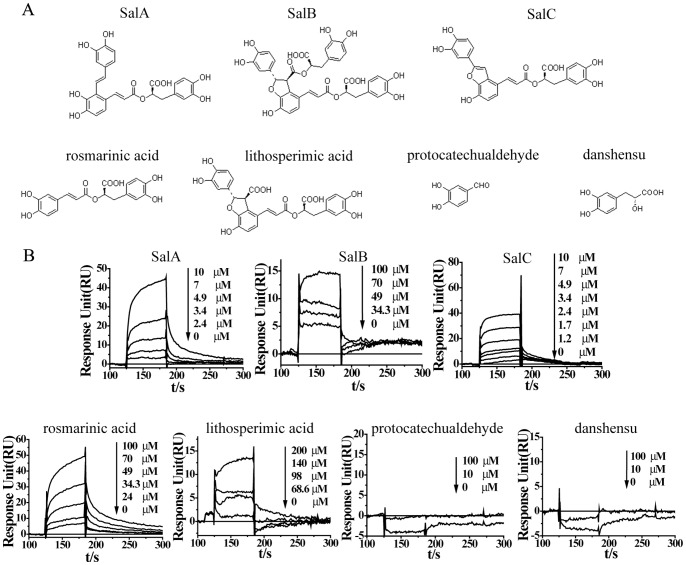
SalA binds with MMP-9 directly. (**A**) Chemical structure of seven representative phenolic acids from *Salvia miltiorrhiza.* Four types of phenolic acids were identified based on the number of benzene rings in their structure, including monomer (protocatechualdehyde and danshensu), dimer (rosmarinic acid), trimer (SalA, SalC, lithospermic acid) and tetramer (SalB). (B) Direct binding of phenolic acids to MMP-9 detected by BIAcore analysis. Lowest equilibrium dissociation constant (K_D_) was found for SalA (0.80 µmol/L), indicating the strongest binding of SalA with MMP-9 among 7 phenolic acids.

Four types of phenolic acids from *Salvia miltiorrhiza,* monomer (protocatechualdehyde and danshensu), dimer (rosmarinic acid), trimer (SalA, SalC, lithospermic acid) and tetramer (SalB) based on the number of benzene rings in their structure were presented (Fig. 1A). Using MMP-9 CD, surface plasmon resonance analysis was performed to measure the interaction between active ingredient and MMP-9 CD. The response was proportional to the ingredient concentration. Using steady state affinity fit model to determine the equilibrium dissociation constant (K_D_), it was calculated that the K_D_ value was 0.80 µmol/L (SalA), 32.9 µmol/L (SalB), 1.13 µmol/L (SalC), 10.7 µmol/L (Rosmarinic acid), 66.2 µmol/L (lithospermic acid), suggesting the strongest binding of SalA with MMP-9 among 7 phenolic acids (Fig. 1B).

### Competitive Inhibition of SalA on MMP-9

**Figure 2:**
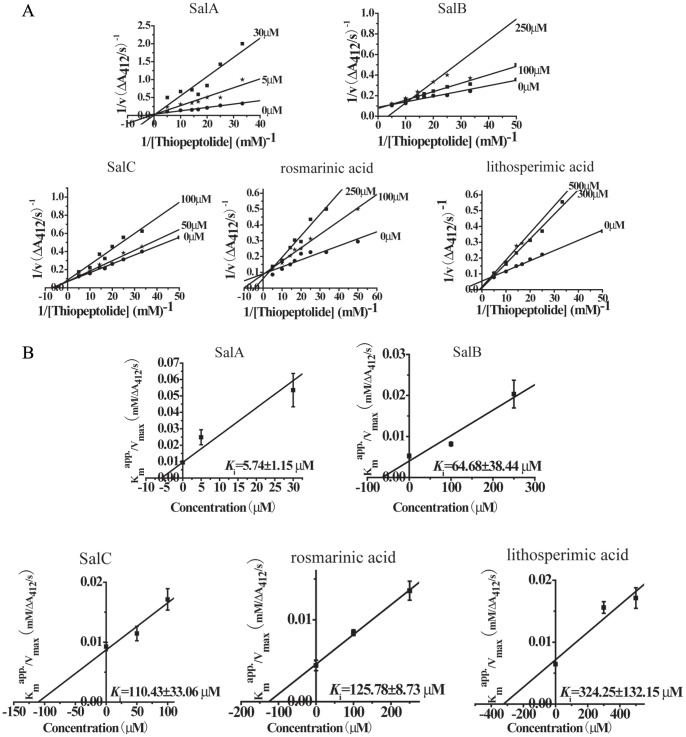
SalA inhibits MMP-9 activity competitively. (A) Kinetic analysis of phenolic acids against MMP-9 CD through double reciprocal plots of 1/V versus 1/[thiopeptolide]. (B). Secondary plot of K_m_^app^/V_max_ versus different concentration of compounds.

Direct and competitive inhibition of ingredients on MMP-9 CD was investigated by enzyme kinetic analysis. The double-reciprocal plots were obtained in the presence of various concentrations of thiopeptolide with or without ingredients. The inhibition mode appeared to be competitive, with the characteristics of intersecting lines with different 1/v and 1/[thiopeptolide] axis-intercepts and different slopes (Fig. 2A). Spectrophotometric enzyme kinetic assay revealed that the calculated *K*_i_ value on MMP-9 CD was 5.74±1.15 µmol/L (SalA), 64.68±38.44 µmol/L (SalB), 110.43±33.06 µmol/L (SalC), 125.78±8.73 µmol/L (rosmarinic acid) and 324.25±132.15 µmol/L (lithospermic acid), suggesting the strongest inhibition of SalA on MMP-9 (Fig. 2B). Neither binding nor inhibition was found for protocatechualdehyde and danshensu on MMP-9 CD.

### SalA Inhibits Neonatal Cardiac Fibroblast Migration Induced by MMP-9 CD

**Figure 3:**
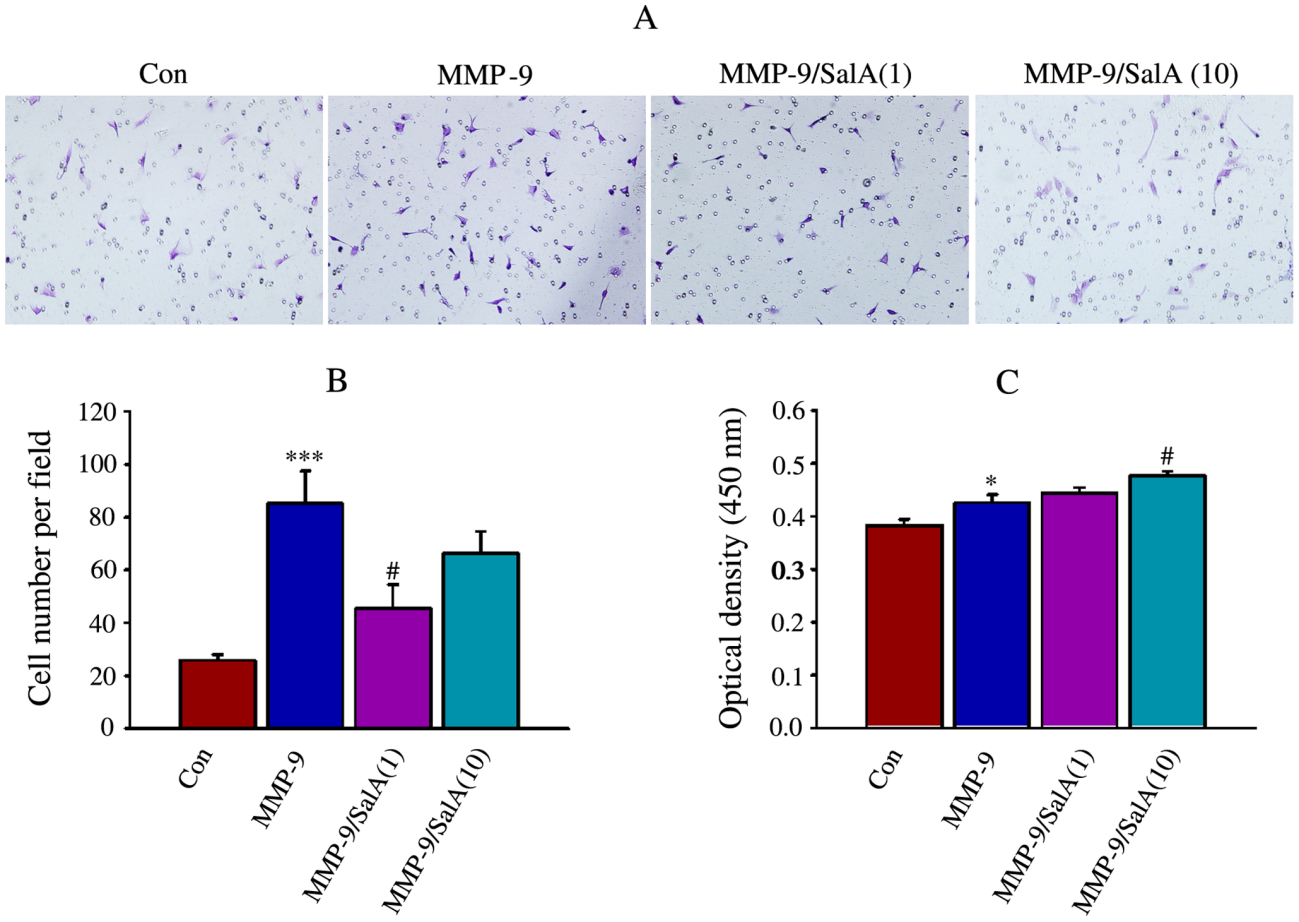
SalA inhibited cardiac fibroblast migration induced by MMP-9 CD. (A) The representative pictures for migration of cardiac fibroblast detected by transwell migration assay, the magnification was 200 X. (B) The quantitative data for migration of cardiac fibroblast. (C) Proliferation of cardiac fibroblast detected by CCK-8. Triplicate determinations were performed for each experimental condition and data are expressed as mean±SE. *p<0.05, ***p<0.001 versus Con; #*p*<0.05 versus MMP-9.

The inhibitory effects of SalA on MMP-9 induced migration of cardiac fibroblasts were examined using transwell assay and the representative pictures are shown (Fig. 3A). Incubation with MMP-9 significantly up-regulated cell migration (85.2±12.4 cell per field) compared to Con (25.9±2.1 cells per field). Treatment with SalA reversed the increase of cell migration induced by MMP-9 significantly, and only about half of the migrated cells were detected in wells treated with 1 µmol/L SalA (45.3±9.1 cells per field) (Fig. 3B).

To elucidate inhibition of SalA on cell migration related with its effects on proliferation or not, the proliferation of neonatal cardiac fibroblast was detected by CCK-8 assay. Significant induction of MMP-9 CD on proliferation was found and 10 µmol/L SalA showed considerable promotion on MMP-9 CD (Fig. 3C), suggesting that the inhibition of SalA on migration was not relative with its effects on proliferation.

### SalA Inhibited the Transition of Cardiac Fibroblast to Myofibroblast Induced by MMP-9

**Figure 4:**
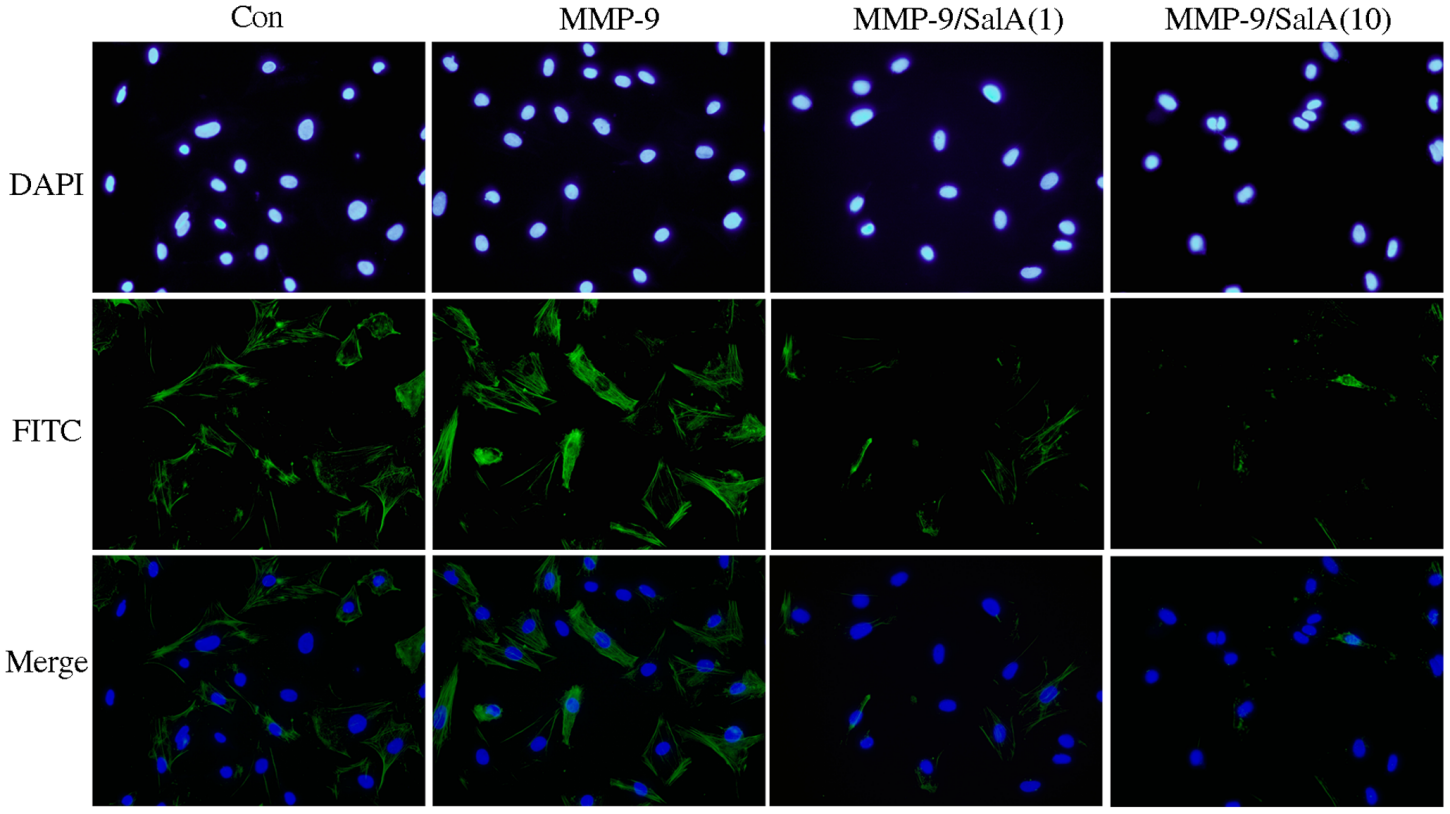
SalA inhibited myofibroblast transformation induced by MMP-9 CD. Fluorescence immunohistochemistry, using a specific α-SMA first antibody following by a second antibody conjugated with FITC, was performed to demonstrate myofibroblast transformation induced by MMP-9. Nuclei were stained with DAPI and images are shown at 400x. MMP-9/SalA(1) or MMP-9/SalA(10) denoted 1 µmol/L SalA or 10 µmol/L SalA was used to detect the inhibitory effect of SalA on MMP-9.

Fibroblasts may respond to mechanical loading by a switch to a myofibroblastic phenotype wherein they express α-SMA [16]. Myofibroblasts transformation from cardiac fibroblasts is a critical event in the initiation of myocardial fibrosis. To detect the effects of SalA on myofibroblast transformation induced by MMP-9, the total cells were identified by DAPI stain, and the α-SMA positive cells were identified by FITC stain. In the present study, up-regulation of α-SMA was detected by immunofluorescence of FITC in MMP-9 CD treated cells, and SalA reversed the effects of MMP-9 CD (Fig. 4).

### SalA Inhibited Cytokine Secretion and Collagen Synthesis of Cardiac Fibroblast Induced by MMP-9

**Figure 5:**
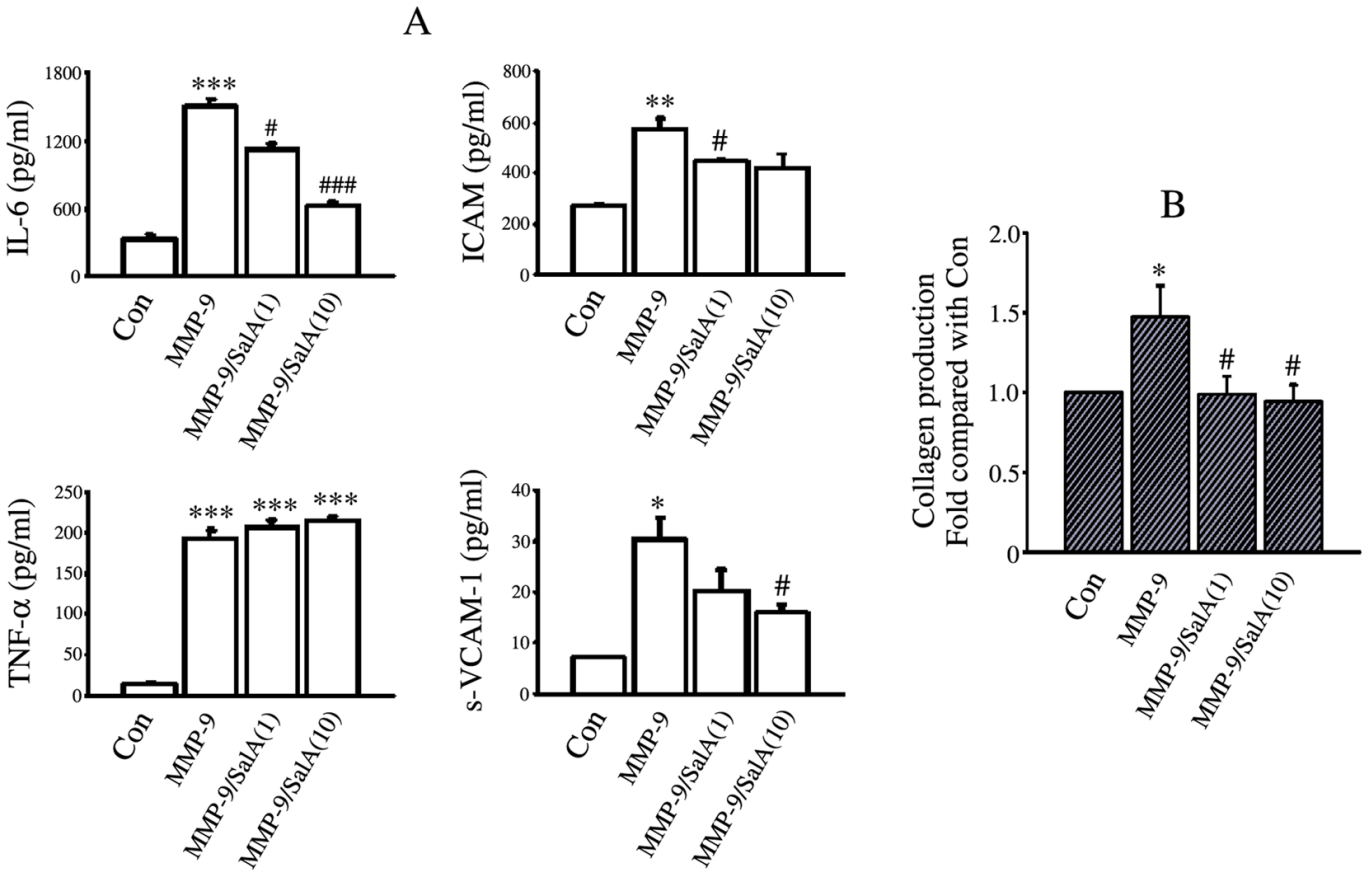
SalA inhibited the secretion of cytokines and collagen induced by MMP-9 CD. (A) MMP-9 CD stimulated the secretion of IL-6, ICAM, sVCAM-1, TNF-α and SalA reversed the effects of MMP-9 CD partially. (B) SalA inhibited collagen secretion induced by MMP-9 CD. MMP-9/SalA(1) or MMP-9/SalA(10) denoted 1 µmol/L SalA or 10 µmol/L SalA was used to detect the inhibitory effects of SalA on MMP-9. *p<0.05, **p<0.01, ***p<0.001 versus Con; #*p*<0.05, ### *p*<0.001 versus MMP-9.

Three cytokines that are known to facilitate migration of fibroblast were up-regulated by MMP-9 CD, they were IL-6 (*p*<0.001), ICAM (*p*<0.01) and sVCAM-1 (*p*<0.05). Conversely, SalA led to an inhibition on these three cytokines secretion induced by MMP-9 CD. No significant inhibitory effect of SalA on TNF-α secretion induced by MMP-9CD was found (Fig. 5A).

As collagen is a key component of the extracellular matrix regulated by myocardial fibroblasts, we also evaluated collagen secretion from cardiac fibroblasts into the culture medium. There was an about 1.5 fold increase in total collagen production from cardiac fibroblasts after 24 h MMP-9 CD treatment (*p*<0.05). Both 1 µmol/L SalA and 10 µmol/LSalA significantly attenuated net collagen production in the presence of MMP-9 CD (*p*<0.05; Fig. 5B).

### Inhibition of SalA on H9c2 Cells Overexpressing MMP-9

**Figure 6:**
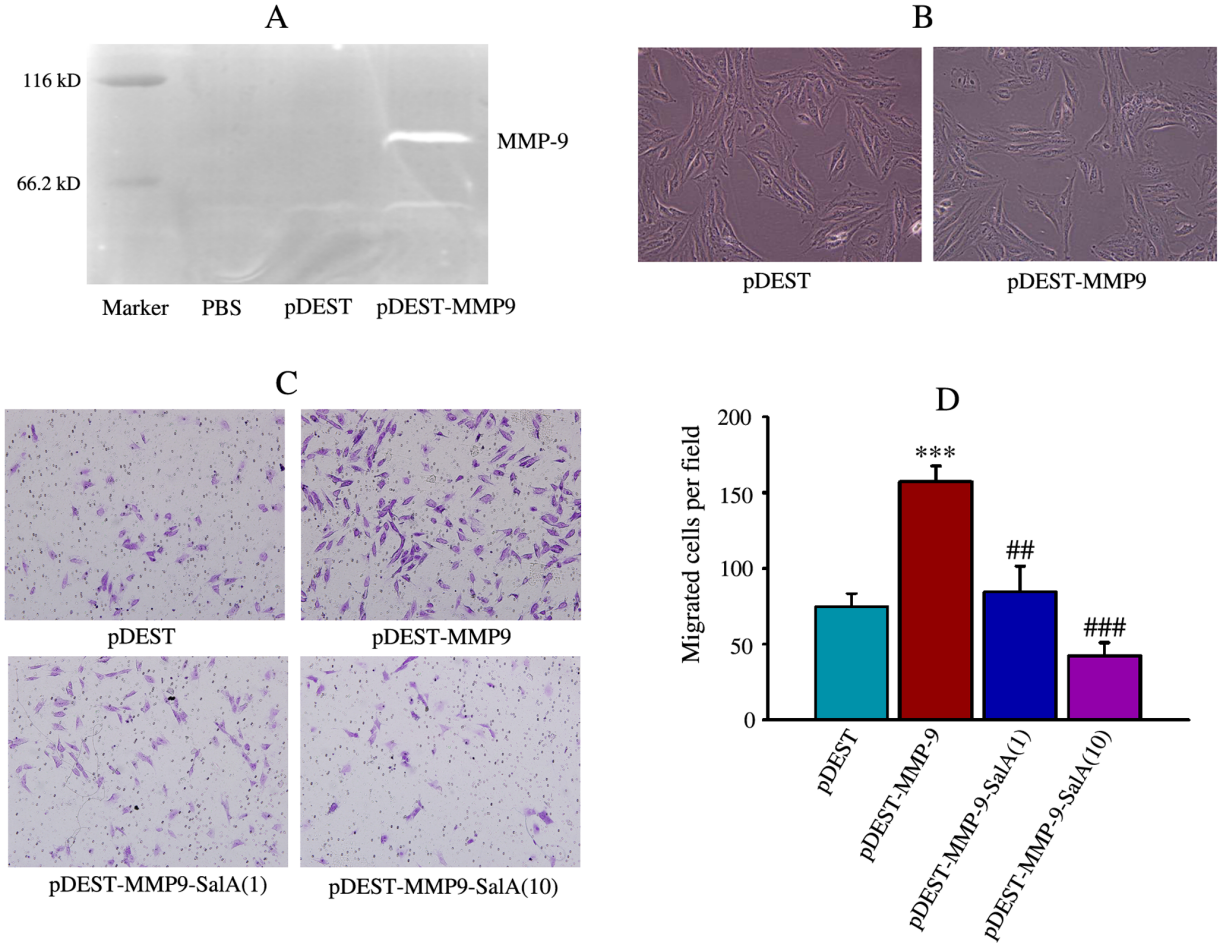
SalA inhibited migration of H9c2 cells with MMP-9 overexpression. (A) Overexpression of MMP-9 was confirmed by gelatin zymography in MMP-9 vector (pDEST-MMP9) transfected H9c2 cells. (B) There are no significant differences on the morphology of empty vector (pDEST), MMP-9 vector (pDEST-MMP9) transfected H9c2 cells. (C) The representative pictures for migration of H9c2 cells stained with crystal violet. (D) The quantitative data for invasion of cells. ***p<0.001 versus pDEST control; ##*p*<0.01, ###*p*<0.001 versus pDEST-MMP-9.

A stable H9c2 cell lines overexpressing MMP-9 was set up and expression of MMP-9 was detected by in-gel zymography (Fig. 6A). There was no significant difference on cell morphology between pDEST transfected control cells and pDEST-MMP9 transfected cells (Fig. 6B). Cell migration was used to confirm the inhibitory effects of SalA on MMP-9 function. Migration of cardiac fibroblasts was examined using transwell assay and the representative pictures were shown (Fig. 6C). Overexpression of MMP-9 significantly increased cell migration comparing with pDEST (157.4±10.2 versus 74.6±8.9 cells per field), and SalA significantly reversed this increase (84.4±17.0 versus 157.4±10.2 cells per field for 1 µmol/L SalA; 42.3±8.9 versus 157.4±10.2 cells per field for 10 µmol/L SalA) (Fig. 6D).

### SalA Down-regulated MMP-9 Activity, Improved Cardiac Fibrosis and Hypertrophy of SHRs

**Figure 7:**
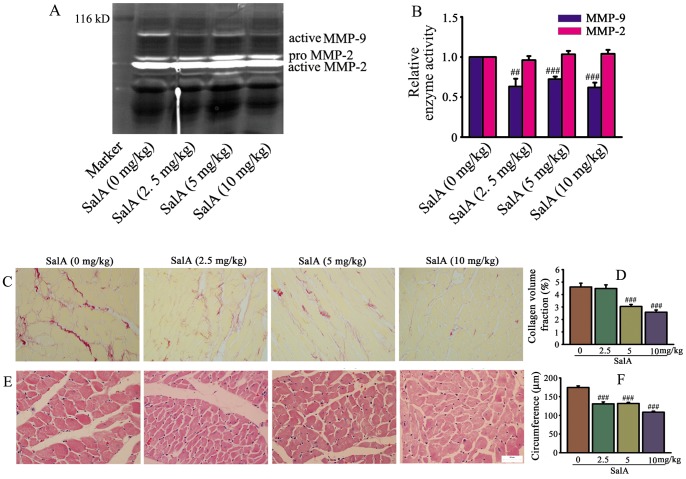
SalA inhibited MMP-9 activity and ameliorated cardiac remodeling in SHRs. (A) The representative zymogram for MMP-9 and MMP-2 enzymatic activities in each treatment group *in vivo*. (B) Quantification of MMP-9, MMP-2 activity expressed as fold decrease versus SalA (0 mg/kg). (C) Representative area of whole heart stained by Sirius red. The position of collagen deposition was stained in red. (D) Quantitative data of collagen volume fraction for C. (E) Representative images of heart stained by haematoxylin and eosin. (F) Quantification of cardiomyocyte circumference with different treatment for E. Results are expressed as mean±S.E. #<0.05, ##*p*<0.01, ###*p*<0.001 versus SalA (0 mg/kg).

To detect the inhibition of SalA on gelatinases *in vivo*, we evaluated the activity of MMP-2 and MMP-9 in heart of SHRs using in-gel gelatin zymography (Fig. 7A). MMP-9 activity was inhibited by SalA significantly, while no regulation of SalA on MMP-2 activity was found (Fig. 7B). Very thick, dark stain of collagen fiber was readily visible on rats without SalA treatment; while collagen fiber became thinner, weaker and more discontinuous with increasing dose of SalA (Fig. 7C). The collagen volume fractions (%) of different group were 4.61±0.31 (0 mg/kg); 4.48±0.29 (2.5 mg/kg); 3.04±0.16 (5 mg/kg); 2.58±0.17 (10 mg/kg), respectively (Fig. 7D). Hypertrophy of cardiomyocyte was assessed by myocyte circumference, SalA treatment significantly down-regulated cardiomyocyte size compared to SHR (*p*<0.001, Fig. 7E, F). In consistent with the results of cardiomyocyte size, the protection of SalA against cardiac hypertrophy was further verified by heart weight. The HW/BW (mg/g) was 4.37±0.08 (0 mg/kg), 4.36±0.05 (2.5 mg/kg), 4.37±0.08 (5 mg/kg) and 4.10±0.08 (10 mg/kg), respectively (Table 1).

**Table 1:** Effects of SalA on basic cardiovascular characteristics of SHR.

Parameters	SalA-0 mg	SalA-2.5 mg	SalA-5 mg	SalA-10 mg
BW (g)	318.8±5.5	329.1±7.0	333.3±7.3	317.2±5.1
HW/BW (mg/g)	4.37±0.08	4.36±0.05	4.37±0.08	4.10±0.08#
LW/BW (mg/g)	3.91±0.10	3.71±0.09	3.55±0.08#	3.83±0.06
KW/BW (mg/g)	4.00±0.07	4.00±0.10	4.03±0.10	4.19±0.10

All the values are expressed as mean ± S.E. Heart weight divided by body weight (HW/BW), lung weight divided by body weight (LW/BW) and kidney weight divided by body weight (KW/BW) were also calculated. #*p*<0.05 versus SalA (0 mg/kg). n = 20 per each group. Results are expressed as mean±S.E.

### Toxicological Evaluation of SalA on SHR

**Figure 8:**
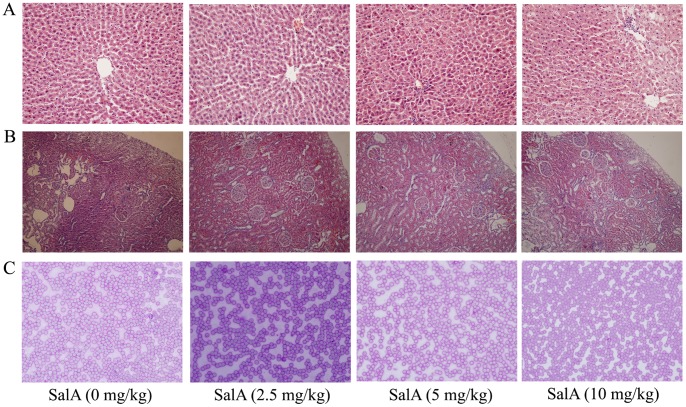
There is no significant toxicity of SalA on SHRs. (A) No significant pathological change was found on liver. (B) No significant pathological change was found on kindey. (C) No significant pathological change was found on blood smear.

As the main component of *Salvia miltiorrhiza*, SalA is considered to be a candidate as a single agent for clinic use. SalA intraperitoneolly administered to rats for 1 month failed to induce any signs of toxicity or mortality. Up to the dose of 10 mg/kg, no changes in body weight, food consumption were found (Table 1). No significantly pathological changes on liver (Fig. 8A), kidney (Fig. 8B) and blood (Fig. 8C) were found.

### A Model for the Mechanism of SalA against Cardiac Remodeling

**Figure 9:**
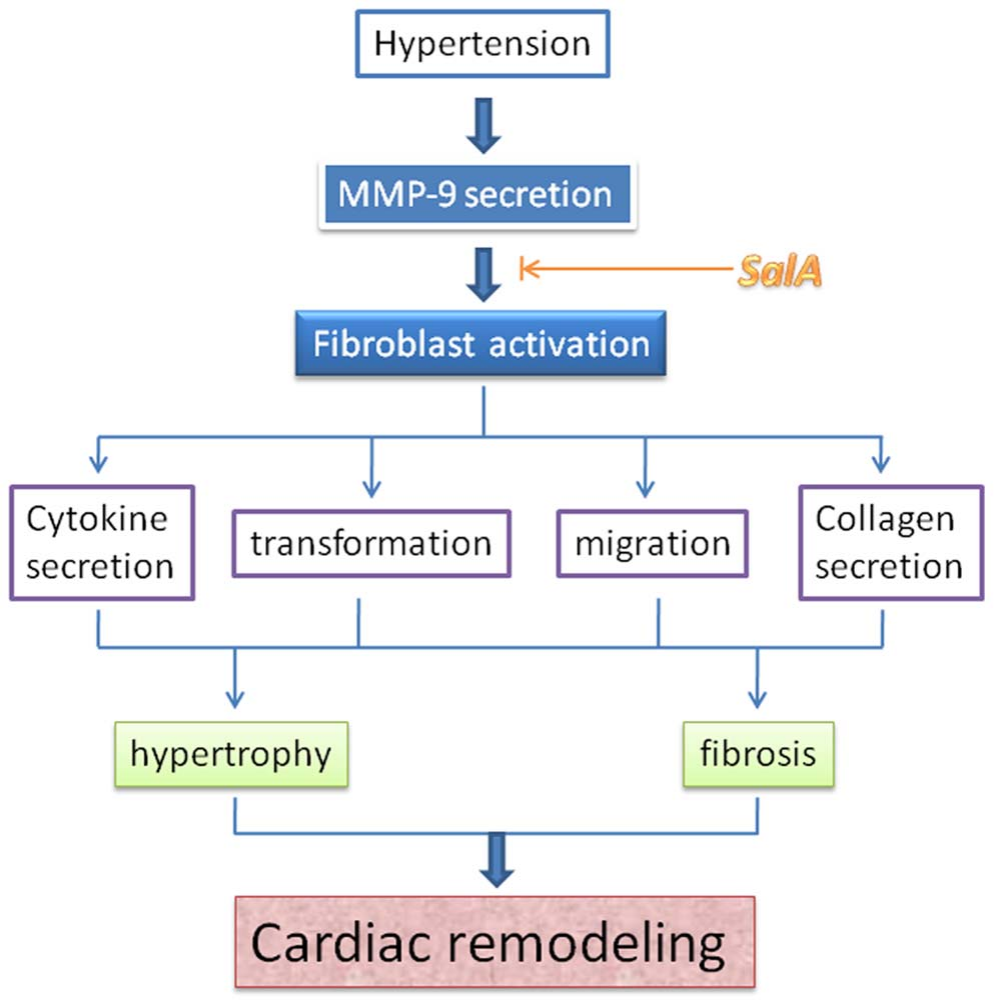
A model for the protective effects of SalA against cardiac remodeling.

Hypertension stimulates MMP-9 secretion, and MMP-9 is one of the main candidates to induce cardiac remodeling. Direct binding and inhibition of SalA on MMP-9 were verified by biacore detection, enzymatic kinetic analysis *in vitro* and in heart of SHR *in vivo*. Decreased MMP-9 activity induced by SalA further attenuates cardiac remodeling through inhibition on fibroblast including cytokine secretion, myofibroblast transformation, migration and collagen secretion. These results indicated that SalA is a novel inhibitor of MMP-9, thus playing an inhibitory role in hypertensive fibrosis (Fig. 9).

## Discussion

Our study indicated that SalA was the most effective MMP-9 inhibitor among seven representative phenolic acids *in vitro* and a specific inhibitor on MMP-9 than MMP-2 *in vivo*. SalA significantly down-regulated fibroblast-myofibroblast transformation, accompanying the regulation of SalA on fibroblast function including mobility, cytokine and collagen secretion. Furthermore, the cardio-protective effects of SalA on SHRs provide sufficient evidence to support the development of SalA as a novel MMP-9 inhibitor against cardiac fibrosis, abnormalities induced by hypertension.

Cardiac fibroblasts account for two thirds of the myocardial cells and play a critical role in maintenance of normal cardiac function including the production and deposition of the majority of ECM proteins in the cardiac interstitium [17], [18]. Improved understanding of how this system is dysregulated in hypertensive heart disease will provide new insights in heart failure and preventive strategies. The fibroblast to myofibroblast transformation is an important event in the development of cardiac fibrosis and scar formation [19]. In the present study, SalA showed the significant inhibition on fibroblast migration, cytokine and collagen secretion, and myofibroblast transformation induced by MMP-9 CD. These results indicate that the antifibrotic activity of SalA is closely related to its inhibition on fibroblast activation.

Hypertension has been associated with elevated levels of angiotensin II, which are well known to directly stimulate cardiac fibroblast proliferation and differentiation into activated myofibroblasts [20]. These myofibroblasts, which characterized by the expression of α-SMA, secrete amount of collagen and contribute to the development of fibrosis [21]. At present study, MMP-9 was recognized as another inducer to directly promote the myofibroblast transformation in cultured fibroblast, and SalA blocked profibrotic signaling and consequently fibroblast differentiation. Direct evidence for the role of MMP systems in pressure overload-induced LV hypertrophy and heart failure suggest that the use of MMP-inhibiters might preserve cardiac function in LV pressure overloading [22]. In agreement with this concept, SalA, as a novel MMP-9 inhibitor, ameliorates cardiac fibrosis and hypertrophy in SHR, indicating MMP-9 inhibitor is promising in the treatment of hypertensive fibrosis.

More than 60 MMP inhibitors with different chemical classes have been in development for treating various diseases, such as arthritis, cancer and cardiovascular disease. However, skin discoloration, musculoskeletal syndrome, and reduced mobility were observed as common side effects in patients treated with these MMP inhibitors [23]. In the present study, SalA administered intraperitoneolly to rats for 1 month failed to induce any signs of toxicity or mortality. Up to the dose of 10 mg/kg, no changes in body weight, food consumption, organ ratio, hematological change, or gross pathology of liver and kidney were found. *Salvia miltiorrhiza* has been used in clinic in China for thousands of years to treat heart failure with little side effects. As the main component of *Salvia miltiorrhiza,* SalA and its analogues hold significant potential for new MMP-9 inhibitor development.

In summary, SalA exhibits the strongest inhibition on MMP-9 among seven phenolic acids from *Salvia miltiorrhiza* and show cardiac protection against interstitial fibrosis through regulation on fibroblast function. The unique structure of SalA and its relative potency on MMP-9 inhibition afford a great deal of potential for further optimization of this natural compound into therapeutics for cardiovascular disease.

## Supporting Information

Figure S1 **Purity of representative phenolic acids.** Representative HPLC chromatograms of SalA, SalB, SalC, rosmarinic acid, lithosperimic acid, protocatechualdehyde, danshensu. The purity of every compound was more than 99%.
(TIF)

Figure S2 **Characteristics of recombinant MMP-9 CD. (A)** Representative structural domains of MMP-9. **(B)** The peptide sequence of recombinant MMP-9 catalytic domain (MMP-9 CD). **(C)** High purity of MMP-9 CD was verified as a single band on 10% SDS-PAGE. **(D)** MMP-9 **CD** displayed significant gelatinase activity detected by zymography.
(TIF)
