# Supplementary figures and images for: Salvianolic Acid A, a Novel Matrix Metalloproteinase-9 Inhibitor, Prevents Cardiac Remodeling in Spontaneously Hypertensive Rats
Source: PLoS One. 2013 Mar 22;8(3):e59621. doi: 10.1371/journal.pone.0059621 (PMC3606118; doi:10.1371/journal.pone.0059621)

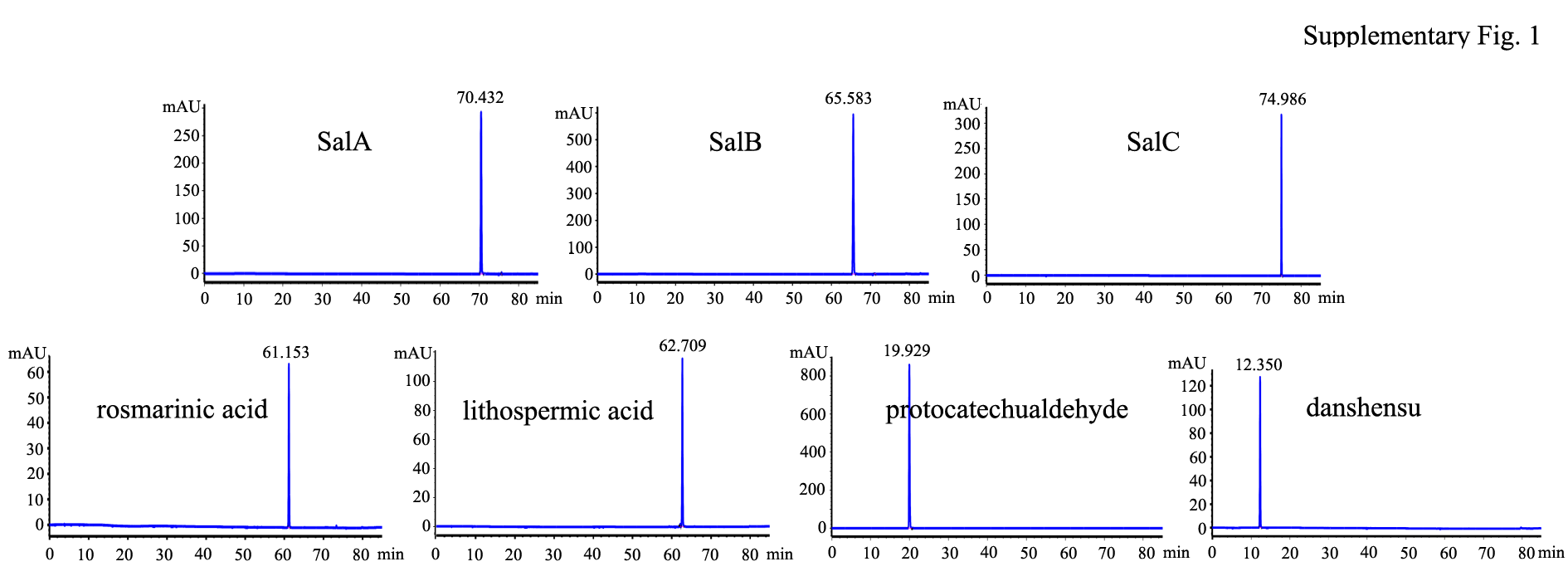

Supplement: Figure S1 — Purity of representative phenolic acids. Representative HPLC chromatograms of SalA, SalB, SalC, rosmarinic acid, lithosperimic acid, protocatechualdehyde, danshensu. The purity of every compound was more than 99%. (TIF) [file pone.0059621.s001.tif]

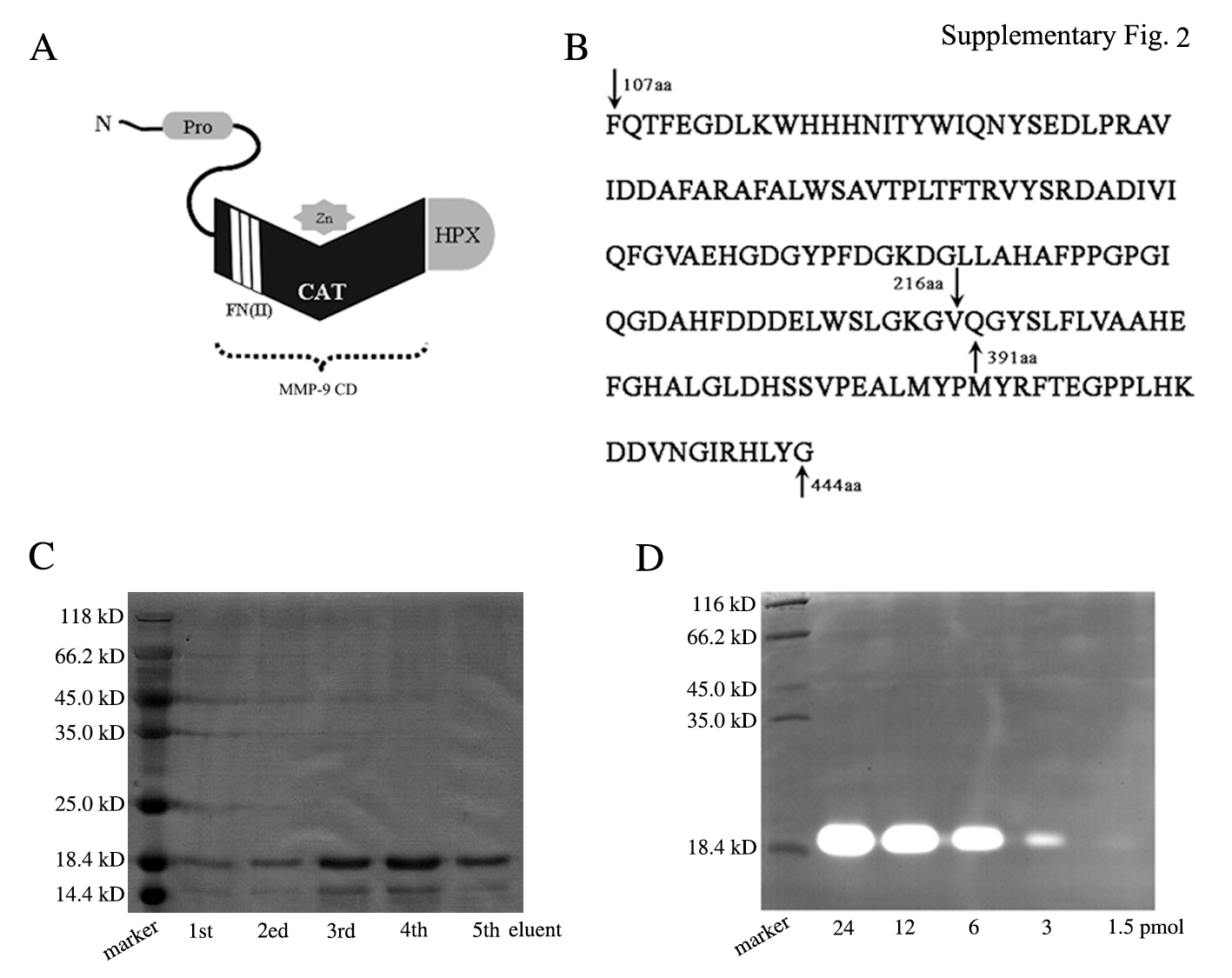

Supplement: Figure S2 — Characteristics of recombinant MMP-9 CD. (A) Representative structural domains of MMP-9. (B) The peptide sequence of recombinant MMP-9 catalytic domain (MMP-9 CD). (C) High purity of MMP-9 CD was verified as a single band on 10% SDS-PAGE. (D) MMP-9 CD displayed significant gelatinase activity detected by zymography. (TIF) [file pone.0059621.s002.tif]
